# Impacts of changes in vegetation on saturated hydraulic conductivity of soil in subtropical forests

**DOI:** 10.1038/s41598-019-44921-w

**Published:** 2019-06-10

**Authors:** Mingzhuo Hao, Jinchi Zhang, Miaojing Meng, Han Y. H. Chen, Xiaoping Guo, Shenglong Liu, Lixin Ye

**Affiliations:** 1 Jiangsu Province Key Laboratory of Soil and Water Conservation and Ecological Restoration, Co-Innovation Center for the Sustainable Forestry in Southern China, College of Forestry, Nanjing Forestry University, 159 Longpan Road, Nanjing, Jiangsu 210037 China; 2 Faculty of Natural Resource Management, Lakehead University, 955 Oliver Road, Thunder Bay, Ontario P7B 5E1 Canada; 3 Nanjing Institute of Environmental Sciences, Ministry of Ecology and Environment, 8 Jiangwangmiao Street, Nanjing, Jiangsu 210042 China; 4 Feng yang Mountain Administration of Zhejiang Feng yang Mountain-Baishanzu National Nature Reserve, 55 Zhongshan West Road, Longquan, Zhejiang 323700 China

**Keywords:** Forest ecology, Hydrology

## Abstract

Saturated hydraulic conductivity (*K*_*s*_) is one of the most important soil properties that determines water flow behavior in terrestrial ecosystems. However, the *K*_*s*_ of forest soils is difficult to predict due to multiple interactions, such as anthropological and geomorphic processes. In this study, we examined the impacts of vegetation type on *K*_*s*_ and associated mechanisms. We found that *K*_*s*_ differed with vegetation type and soil depth, and the impact of vegetation type on *K*_*s*_ was dependent on soil depth. *K*_*s*_ did not differ among vegetation types at soil depths of 0–10 and 20–30 cm, but was significantly lower in managed forest types (mixed evergreen broad-leaved and coniferous forests, bamboo forests, and tea gardens) than native evergreen broadleaf forests at a depth of 10–20 cm. Boosted regression tree analysis indicated that total porosity, non-capillary porosity, and macro water-stable aggregates were the primary factors that influenced *K*_*s*_. Our results suggested that vegetation type was a key factor that influences hydraulic properties in subtropical forest soils through the alteration of soil properties, such as porosity and macro water-stable aggregates.

## Introduction

Saturated hydraulic conductivity (*K*_*s*_) is one of the most important soil properties that determines the behavior of water flow systems^1^. A detailed understanding of *K*_*s*_ is critical in the assessment of irrigation practices, infiltration rates, runoff, groundwater recharge rates, and drainage processes, which makes it of particular concern in forest management^2^.

Vegetation is expected to be an important factor that influences the hydraulic properties of soil by affecting its physical and chemical characteristics^3,4^. Forest conversion is a major change globally, yet our understanding of its impacts on soil *K*_*s*_ remains incomplete. However, the prediction of forest soil *K*_*s*_ is complex due to multiple interactions associated with anthropological and geomorphic processes, which impact spatiotemporal *K*_*s*_ variations^5–7^. Previous studies have found differences in *K*_*s*_ among deforested areas in primary forests, secondary forests, and agricultural ecosystems^8^, and among forests, shrub lands, and grasslands^9^. Intense agricultural use can reduce *K*_*s*_ soils^10^. Pasture soils have lower *K*_*s*_ than woodland soils^11^. The mechanisms responsible for *K*_*s*_ are associated with soil structure^12^. Across soil depth profiles, *K*_*s*_ tends to decrease with soil depth^13–16^. This elucidation has been integrated within several hydraulic models^17,18^, in which pedotransfer functions (PTF) models are typically applied to the prediction of *K*_*s*_^5,19^. Among a number of physical parameters, soil porosity, texture, and bulk density are determinants for *K*_*s*_ in the PTF^20–23^. The chemical characteristics of soils such as soil organic carbon (SOC) or soil organic matter (SOM) are also important predictors for *K*_*s*_ in the PTF^21,24–26^. The effects of soil aggregate dimensions on *K*_*s*_ were investigated and it was found that higher *K*_*s*_ associated with higher SOM was positively associated with soil aggregate size^27^. *K*_*s*_ has also been found to be affected by pore dimensions and distribution^28^. At the field scale, however, the physical and chemical parameters of soils are not always significantly correlated with *K*_*s*_^29^.

Despite the drastic effects of forest vegetation type shifts that occur frequently at a global scale, how changes in forest vegetation types affect *K*_*s*_ remains poorly understood. Moreover, the contribution and importance of specific soil parameters on the resulting *K*_*s*_ is not always certain. Here, our objectives are to test: (1) whether differences in forest vegetation, resulting from changes in management objectives, affect soil *K*_*s*_ across multiple soil depths and; (2) how changes in soil *K*_*s*_ might be associated with the physicochemical attributes of soil. To address our first objective, we used analysis of variance to test the effect of forest vegetation types on soil *K*_*s*_. For the second objective, we used boosted regression tree (BRT) analysis, which resembles an additive regression model and can achieve higher accuracy and less bias in predictions than traditional multiple regression models^30^. In particular, BRT analysis is good for handling multi-collinearity concerns and violations of parametric assumptions^31,32^.

## Results

**Figure 1:**
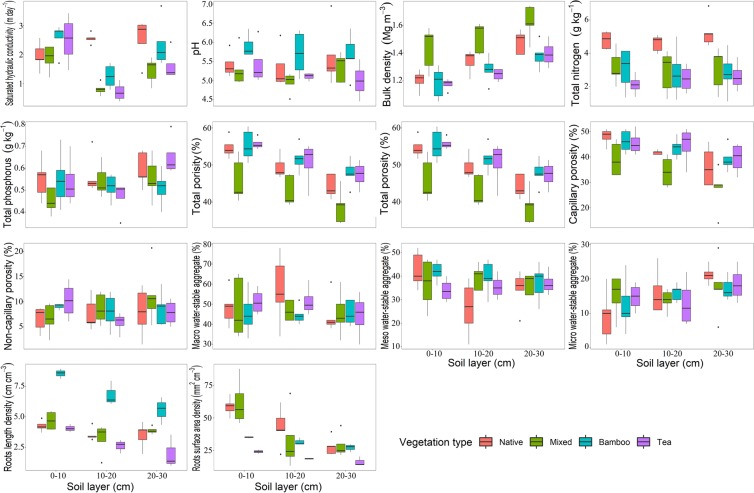
Soil saturated hydraulic conductivity (*K*_*s*_), soil physical and chemical characteristics and roots distribution characteristics in relation to vegetation type and soil depth. Values for boxplots are medians, 75% observations in boxes, and whiskers above and below the box indicate 95^th^ and 5^th^ percentiles. Corresponding statistical analysis results are presented in Table 1.

Both vegetation type and soil depth affected soil *K*_*s*_ and other characteristics (Table 1). The effect of vegetation type on *K*_*s*_ was significantly dependent on soil depth as indicated by the significant interaction effect between vegetation type and soil depth (*P* = 0.03). In the top soil layer (0–10 cm), soil *K*_*s*_ was higher in bamboo forests and tea gardens than in native and mixed forests; in the deep soil layers (10–20 cm and 20–30 cm), native forests had significantly higher soil *K*_*s*_ than other vegetation types, with the lowest values in tea gardens (Fig. 1).

**Table 1:** Effects of vegetation type and soil depth on soil saturated hydraulic conductivity (*K*_*s*_), soil physicochemical characteristics, and roots characteristics.

	Vegetation type (*df* = 3)		Soil depth (*df* = 2)		vegetation type × Soil depth (*df* = 6)	
	*F*	*P*	*F*	*P*	*F*	*P*
*K*_*s*_ (m day^−1^)	9.22	<0.001	1.53	0.23	2.65	0.03
Bulk density (Mg m^−3^)	20.24	<0.001	8.16	<0.001	0.20	0.97
PH	0.82	0.52	1.24	0.31	0.47	0.82
Total organic carbon (g kg^−1^)	0.45	0.72	1.73	0.19	0.49	0.81
Total nitrogen (g kg^−1^)	4.10	0.01	0.34	0.72	0.21	0.97
Total phosphorus (g kg^−1^)	1.21	0.32	1.32	0.28	1.56	0.18
Total porosity (%)	16.93	<0.001	6.93	<0.001	0.18	0.98
Capillary porosity_trans_ (%)	11.26	<0.001	6.23	<0.001	0.17	0.98
Non-capillary porosity_trans_ (%)	1.21	0.32	1.43	0.25	1.61	0.17
Macro water-stable aggregate (%)	1.27	0.30	0.78	0.47	0.73	0.63
Meso water-stable aggregate (%)	1.79	0.16	3.56	0.04	1.77	0.13
Micro water-stable aggregate (%)	0.39	0.76	4.57	0.02	0.74	0.62
Roots length density (cm cm^−3^)	19.71	<0.001	19.36	<0.001	2.85	0.04
Roots surface area density (mm^2^ cm^−3^)	4.38	0.04	8.79	<0.001	0.79	0.59

Capillary porosity_trans_ – Transformed capillary porosity by Box-Cox transformation with λ = 2.4; Non-capillary porosity_trans_ – Transformed Non-capillary porosity Box-Cox transformation with λ = 0.55.

The soil bulk density was impacted by changes in vegetation type and soil depth, but not by the interaction between vegetation type and soil depth (*P* < 0.001, *P* < 0.001 and *P* = 0.97, respectively). A similar trend was seen for total porosity and capillary porosity, which were impacted by vegetation type and soil depth, but not by their interaction. Total soil nitrogen was impacted considerably by changes in vegetation type with *P* = 0.01, while the impacts of soil depth and the interaction between vegetation type and soil depth were not significant. The non-capillary porosity of soil was impacted by the interaction between vegetation type and soil depth with *P* = 0.09, but not by these individual factors (*P* = 0.21 and *P* = 0.36, respectively). Meso and micro water-stable aggregates were impacted only by soil depth with *P* = 0.04 and *P* = 0.02, respectively (Table 1, Fig. 1).

Both root length density and root surface area density varied across vegetation types and soil depths. Root length density was impacted by changes in vegetation type, soil depth, and the interaction between them (*P* < 0.001, *P* < 0.001 and *P* = 0.04, respectively). Root surface area density was impacted by changes in vegetation type and soil depth (*P* = 0.04 and *P* < 0.001, respectively), but not by their interaction (P = 0.59). Root length density of bamboo forests was higher than other vegetation types in all soil depths, while root surface area density was lower in 0–10 cm soil depth. The root length density of bamboo forests in 0–10 cm and 10–30 cm was lower (Table 1, Fig. 1).

**Figure 2:**
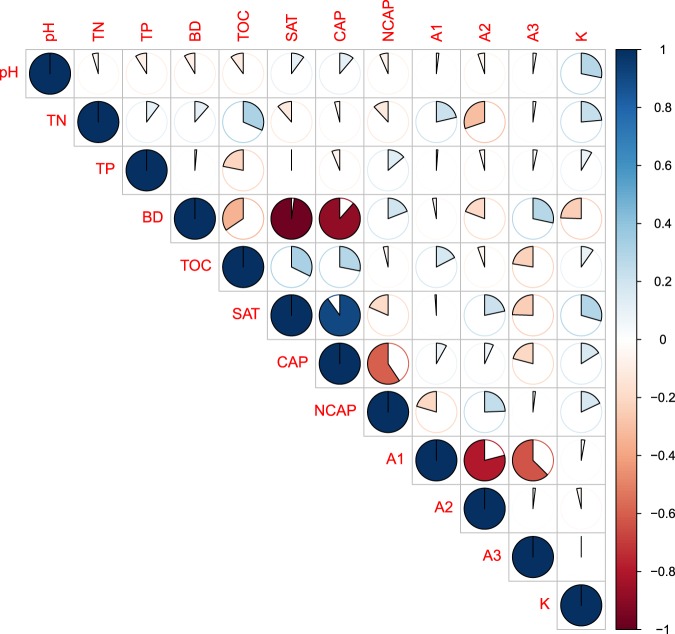
Correlation between saturated hydraulic conductivity (*K*_*s*_) and soil characteristics.

**Figure 3:**
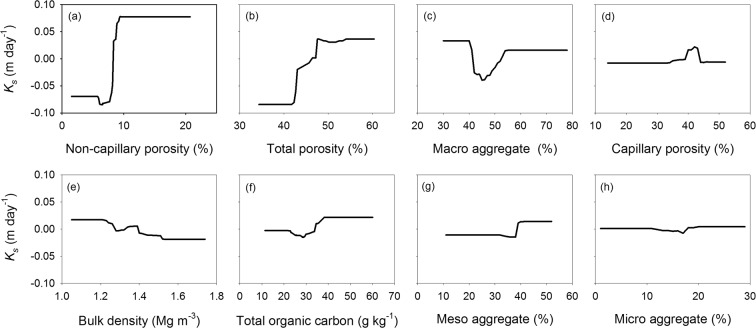
Boosted regression tree (BRT) modeled relationships between saturated hydraulic conductivity (*K*_*s*_) (centered value, m day^−1^) and (**a**) non-capillary porosity (%), (**b**) total porosity, (**c**) macro water-stable aggregate content (%), (**d**) capillary porosity (%), (**e**) bulk density (Mg m^−3^), (**f**) total organic carbon (g kg^−1^), (**g**) meso water-stable aggregate content (%), and (**h**) micro water-stable aggregate content (%).

**Figure 4:**
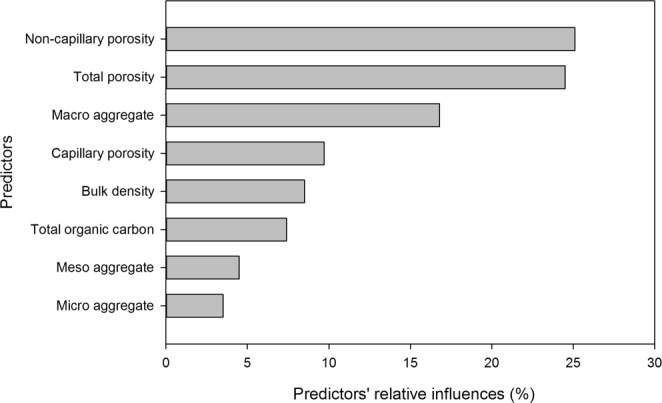
Results of boosted regression tree analysis (BRT) of predictors’ relative influences (%).

Correlation analysis showed the correlation between all soil properties (Fig. 2). All soil properties contributed to differences in *K*_*s*_, with non-capillary porosity, total porosity, and macro water-stable aggregates exhibiting the greatest contributions (Fig. 3). BRT analysis indicated that non-capillary porosity, total porosity, and macro water-stable aggregates contributed 25.1%, 24.5%, and 16.8% of the BRT model explained variations in *K*_*s*_, respectively (Fig. 4). The other factors of capillary porosity, bulk density, total organic carbon, meso water-stable aggregates, and micro water-stable aggregates, had relatively minor contributions in this model (Fig. 4).

## Discussions

Soil *K*_*s*_ is a critical factor for plant growth that involves air-filled porosity, plant-available water, and so forth^33^. Hence, the improvement of *K*_*s*_ is essential in order to avoid runoff and soil erosion^34^. As we anticipated, *K*_*s*_ differed among the four vegetation types, with the disparities resulting primarily at the 10–30 cm soil depth. This result was similar to previous research, in which changes in vegetation type were shown to alter *K*_*s*_ significantly^35^. The effects of vegetation type on soil *K*_*s*_ were probably by means of root distribution and morphological characteristics such as root biomass and distribution in soil^36–39^. The root system affects soil texture via mechanical forces such as insertion or extrusion in soil^40^. Root length density and root surface area density both showed a decreasing trend with respect to depth in different vegetation types. In native forests and mixed forests, the main roots are obvious and the root system might extend to the lower depths of the layer, while in bamboo forests, the main roots are not obvious and the underground rhizome expands near the soil surface. In tea gardens, roots do not extend as deeply in soil compared to the other two types, nor do they expand like bamboo rhizome.

The roots distribution characteristics also affect the soil texture by adjusting litter input from the soil or its surface over time^41^, thus affecting soil organic carbon (SOC) and other soil physicochemical characteristics^3,4^. This stimulates belowground microbial biomass and rhizospheres^42,43^, and the effects of the physicochemical attributes of soil on *K*_*s*_ via microbial community activities^44–46^. So to alter the physiochemical characteristics of soil, by means of producing solid, gas, and gel phases in order to adjust the fraction of the total spatial volume that is available for water flow, and hence the *K*_*s*_.

The effects of soil depth on soil *K*_*s*_ differed with vegetation types though *K*_*s*_ in native forests did not vary with soil depth. This is in alignment with prior researches^13–15^, which may have been the result of distinct vertical distributions of the physicochemical characteristics of soil and root distribution^14^. This might partly explain the increasing trend in soil depth from 10–20 cm to 20–30 cm. The decreasing root length and surface area densities were weakened by the effects of roots via mechanical forces or litter input characteristics. The higher *K*_*s*_ in 0–10 cm might be attributed to the great probability in contacting a fresh litter of leaves or branches.

In this study, the variation of the *K*_*s*_ value was higher in the tea garden. This may be attributed to the higher distribution density of the tea stems and the complexity of the root distribution underground, which could affect the *K*_*s*_ value^28,37^. We also observed that the *K*_*s*_ value at the 10–20 cm depth for the vegetation types other than native forests was lower than at the other soil depths. We attributed this to soil disturbances during vegetation conversion at that time, which might have had the effect of compacting the 0–20 cm depth layer. However, since the relation between *K*_*s*_ and root distribution was not clarified in this study, we propose to explore this area further in future research.

In this study, total soil porosity, non-capillary porosity, and macro water-stable aggregates were the principal factors that influenced *K*_*s*_. A key parameter in this study was bulk density, from which the calculations on total soil porosity were derived. This was similar to a study in which differences in *K*_*s*_ between samples were found to be correlated with bulk density and macro porosity^47^. The characteristics of pores in soils, such as their dimensions, distribution, and interconnections have been known to impact *K*_*s*_. It was found in many studies that lower bulk density was aligned with higher *K*_*s*_, and vice versa^48,49^, while water stable macro aggregates were positively correlated with *K*_*s*_^50^. It was found that the *K*_*s*_ values were reduced in soils with smaller aggregates in contrast to those with large aggregates^12^. This was likely attributed to the impacts of different fresh organic matter, which were produced by different vegetation types^51^. Vegetation generated litter may simulate soil aggregation^52^, which subsequently influences bulk density and porosity^53,54^, while bulk density and porosity are closely correlated to adjustments in *K*_*s*_^55,56^. It remains a scientific challenge to describe in detail the complex continuous soil space^28^. However, we may conclude that soil pore characteristics are important factors.

In conclusion, our results show that change in vegetation type is a driving factor that strongly influences the hydraulic properties of soils in subtropical forests. Vegetation type, soil depth, and their interaction were observed to influence *K*_*s*_ significantly, and the effects of soil depth on *K*_*s*_ varied for different types of vegetation. The *K*_*s*_ of native forests did not significantly differ at soil depths from 0–30 cm. For the other vegetation types, the *K*_*s*_ at the 10–20 cm depth was significantly lower than that at 0–10 cm and 20–30 cm depths. There are multiple factors that impact *K*_*s*_; however, total soil porosity, non-capillary porosity, and macro water-stable aggregates comprised the primary factors in this study. Soil *K*_*s*_ is strongly influenced by changes in vegetation type, indicating that shifts in aboveground vegetation may strongly impact the water dynamics of soil. Based on our data, we suggest that the restoration of the native evergreen broad-leafed forests will assist in the retention and maintenance of soil hydrologic properties. Additional research will be required to confirm other factors and mechanisms that influence *K*_*s*_, such as the role of root systems and microbial communities in the processes that follow changes in vegetation species.

## Materials and Methods

### Study area

Major forest conversion is occurring globally. In China, vegetation change from native evergreen broadleaf forests to mixed evergreen broadleaf and coniferous forests or other vegetation types is common. This study was conducted at the Fengyang Mountain Nature Reserve, Zhejiang Province, China (longitude extending from 119°06′ to 119°15′E, latitude from 27°46′ to 27°58′N, and elevations of from 600 m to 1929 m), which comprised a land area of 15,171 ha. This nature reserve is characterized as having a subtropical climate, with an annual average temperature of 12.3 °C, and annual rainfall of 2,400 mm. Prior to 1970, this area was dominated by native evergreen broad-leaved forests (primarily comprised of *Camellia japonica* Linn., *Cyclobalanopsis glauca* (Thunberg) Oersted, *Eurya japonica* Thunb., and *Rhododendron simsii* Planch.). Intensive selective cutting and reforestation was conducted during 1971–1973, and portions of the forests were converted to mixed evergreen broad-leaved and coniferous forests (henceforth referred to as mixed forests), primarily consisting of *Schima superba* Gardn. et Champ., *Rhododendron simsii*, and *Pinus taiwanensis* Hayata). Subsequent to clear-cutting, pure plantations with *Cunninghamia lanceolata* (Lamb.) Hook., *Cryptomeria fortune* Hooibrenk ex Otto et Dietr., bamboo forests (*Phyllostachys heterocycla* (Carr.) Mitford cv. Pubescens Mazel ex H.de leh.), or tea gardens were established. Following the establishment of the nature reserve in 1975, the entire study area, including the tea gardens, was protected from anthropogenic disturbances. The roots characteristics of different vegetation types were shown in Supplementary Figure.

### Sampling

In June 2013, we randomly sampled five native evergreen broad-leaved forest stands, five mixed forest stands, five bamboo forest stands, and four tea garden stands at elevations ranging from 1250 to 1450 m, which resulted in a total of 19 sampled stands. All of the sample stands resided on well-drained mesic sites with slopes inclines of less than 5% to minimize the effects of inherent site conditions on soil characteristics^57,58^. In each stand, we established a sample plot of 20 × 20 m. Using a knife and a trowel, we extracted soil samples at depths of 0–10 cm, 10–20 cm, and 20–30 cm by digging a 15 × 15 cm section at each sampling point to enable the analysis of soil physicochemical characteristics^59,60^, which resulted in a total of 57 samples. For the determination of soil *K*_*s*_, bulk density, and capillary porosity analysis, we extracted soil samples with a metal corer (5.5 cm in diameter x 5 cm in height) at each sampling point^61^, which resulted in a total of 171 samples (57 samples for *K*_*s*_ analysis, 57 samples for soil bulk density analysis and capillary porosity analysis, and 57 samples for other physicochemical properties).

To further understand how roots affect the impact of vegetation type on *K*_*s*_, we did supplementary sampling of soil with a metal corer (5.5 cm in diameter × 5 cm in height) in December 2018. Similar to the early sampling, we randomly sampled five native evergreen broad-leaved forest stands, four mixed forest stands, three bamboo forest stands, and three tea garden stands at elevations ranging from 1250 to 1450 m, which resulted in a total of 15 supplementary sampled stands.

### Saturated hydraulic conductivity measurements

*K*_*s*_ was determined based on the constant hydraulic head method by imposing a stable hydraulic head to the top of the cores that were sampled at each of the sampling points, which were saturated with water prior to experiments in the laboratory^1^.

### Analysis of soil physicochemical and roots properties

Soil bulk density was determined by drying the samples in an oven at 105 °C until a constant weight was attained, and then adjusting for root and stone volume^58^. Soil samples for other physicochemical analyses were air-dried, sieved (2 mm mesh) in the laboratory, and then stored in air-tight plastic bags. Total organic carbon (TOC) content was measured using the sulfuric acid-potassium external heating method^62^. Total nitrogen and total phosphorus were simultaneously determined using a Bran + Luebbe Autoanalyser 3 Continuous Flow Analyzer (Bran + Luebbe GmbH, Norderstedt, Germany). Root length density and root surface area density were analyzed with the Win RHIZO root system (Regent Instruments, Québec, Canada). Before the analysis, all roots were washed out from the metal corer and then scanned with EPSON LA (Seiko Epson Corporation, Nagano-ken, Japan).

### Soil porosity

The total porosity was calculated using the following equation^63^:

1 $${P}_{t}=100\times (1-{D}_{b}/{D}_{p})$$

where *P*_*t*_ is the total soil porosity (%); 100 is the unit conversion factor; *D*_*b*_ is the soil bulk density (g cm^−1^); and *D*_*p*_ is the soil particle density (g cm^−1^), which was assumed to be 2.65 g cm^−1^ according to China’s standard^64^. The soil capillary porosity was determined based on the water suction method, with the surface of the water located just below the tops of the soil cores^63^. Each soil core was initially weighed and placed onto a salver via filter paper until it attained a constant weight. Following weighing, the soil samples were allowed to drain completely under gravity. The soil samples were subsequently weighed again; their capillary water contents were determined by the differences in weight between the saturated and drained states.

2 $${P}_{c}=100\times {W}_{c}\times {D}_{b}/V$$

3 $${P}_{n}={P}_{t}-{P}_{c}$$

where *P*_*c*_ is the capillary porosity (%); *P*_*n*_ is the non-capillary porosity (%); 100 is the unit conversion factor; *W*_*c*_ is the soil capillary water content (%); *V* is the volume of the soil core (cm^3^).

### Water stable aggregate measurements

Water stable aggregate was measured using a routine wet-sieve method via a mechanical sieving procedure^65^. Briefly, for each soil sample, 200 g of air-dried soil was placed on a series of sieves to determine the dry aggregate size distribution (combined in three nest sizes in the order of >2 mm, 0.5–2 mm, and <0.5 mm) prior to wet-sieving. Subsequently, 50 g samples were prepared according to their dry-sieving percentages by the weight of aggregates at each size distribution for wet-sieving. The samples were immersed in water for 10 minutes and then placed under oscillation at 30 rpm for 30 min. The aggregate fractions that remained on each sieve were removed with aqua distillate into aluminum bins, to be oven-dried at 105 °C for 24 h. The aggregate fractions were then weighed to calculate the aggregate weights from each size class^58,66^.

### Data analysis

To examine the impact of land use type and soil depth on the *K*_*s*_ and other soil characteristics, an analysis of variance (ANOVA) was performed following a split plot design, with soil layers nested within the sample plot. We modelled the fixed effects of vegetation type, soil layer, and their interaction on *K*_*s*_ with plot as the random factor using maximum likelihood with the *lme4* package^67^. ANOVA assumption tests were done with the *lmerTest* package^68^. Shapiro –Wilk’s test was conducted. In this study, the Shapiro –Wilk’s test involving capillary porosity and non-capillary porosity failed, so a Box-Cox transformation was performed by the following equation^69^:

4 $${V}_{trans}=({{V}_{origin}}^{\lambda +1})/\lambda $$

where *V*_*trans*_ is the transformed value of capillary porosity or non-capillary porosity; *V*_*origin*_ is original value of capillary porosity or non-capillary porosity; and *λ* is the parameter of box-cox.

We used boosted regression tree analysis (BRT) to elucidate how *K*_*s*_ was potentially affected by soil physicochemical characteristics. Furthermore, we examined Pearson’s correlation between potential factors and *K*_*s*_ to reduce the fitting predictors. Then we fitted all BRT models using the adjusted settings for ecological modeling: tree complexity = 5, learning rate = 0.0001, bag fraction = 0.7. All analyses were performed using BRT with the R package *gbm*^70^.

## Supplementary information

Supplementary information

## References

[1] Klute, A. & Dirksen, C. Hydraulic conductivity and diffusivity: laboratory methods. *Methods of soil analysis: part 1—physical and mineralogical methods*, 687–734 (1986).

[2] Aimrun W, Amin M, Eltaib S (2004). Effective porosity of paddy soils as an estimation of its saturated hydraulic conductivity. Geoderma.

[3] Breulmann M, Schulz E, Weißhuhn K, Buscot F (2012). Impact of the plant community composition on labile soil organic carbon, soil microbial activity and community structure in semi-natural grassland ecosystems of different productivity. Plant and Soil.

[4] Cookson W (2007). Controls on soil nitrogen cycling and microbial community composition across land use and incubation temperature. Soil Biology and Biochemistry.

[5] Wösten J, Pachepsky Y, Rawls W (2001). Pedotransfer functions: bridging the gap between available basic soil data and missing soil hydraulic characteristics. Journal of Hydrology.

[6] Lohse, K. & Dietrich, W. Contrasting effects of soil development on hydrological properties and flow paths. *Water Resources Research* **41**, 10.1029/2004WR003403 (2005).

[7] Papanicolaou A (2015). Spatial variability of saturated hydraulic conductivity at the hillslope scale: Understanding the role of land management and erosional effect. Geoderma.

[8] Zimmermann B, Elsenbeer H, De Moraes J (2006). The influence of land-use changes on soil hydraulic properties: Implications for runoff generation. Forest Ecology and Management.

[9] Chen X, Zhang Z, Chen X, Shi P (2009). The impact of land use and land cover changes on soil moisture and hydraulic conductivity along the karst hillslopes of southwest China. Environmental Earth Sciences.

[10] Bormann H, Klaassen K (2008). Seasonal and land use dependent variability of soil hydraulic and soil hydrological properties of two Northern German soils. Geoderma.

[11] Zhou X, Lin H, White E (2008). Surface soil hydraulic properties in four soil series under different land uses and their temporal changes. Catena.

[12] Ben-Hur M, Yolcu G, Uysal H, Lado M, Paz A (2009). Soil structure changes: aggregate size and soil texture effects on hydraulic conductivity under different saline and sodic conditions. Soil Research.

[13] Celik I, Ortas I, Kilic S (2004). Effects of compost, mycorrhiza, manure and fertilizer on some physical properties of a Chromoxerert soil. Soil and Tillage Research.

[14] Fu T, Chen H, Zhang W, Nie Y, Wang K (2015). Vertical distribution of soil saturated hydraulic conductivity and its influencing factors in a small karst catchment in Southwest China. Environmental Monitoring and Assessment.

[15] van Lier Q, Wendroth O, van Dam J (2015). Prediction of winter wheat yield with the SWAP model using pedotransfer functions: An evaluation of sensitivity, parameterization and prediction accuracy. Agricultural Water Management.

[16] Wilske B (2015). Effects of Short Term Bioturbation by Common Voles on Biogeochemical Soil Variables. Plos One.

[17] Güntner A, Uhlenbrook S, Seibert J, Leibundgut C (1999). Multi-criterial validation of TOPMODEL in a mountainous catchment. Hydrological Processes.

[18] Hwang T, Band L, Vose J, Tague C (2012). Ecosystem processes at the watershed scale: Hydrologic vegetation gradient as an indicator for lateral hydrologic connectivity of headwater catchments. Water Resources Research.

[19] Bouma, J. In *Advances in Soil Science* (ed. Stewart, B. A.) 177–213 (Springer US, 1989).

[20] Cosby B, Hornberger G, Clapp R, Ginn T (1984). A Statistical Exploration of the Relationships of Soil Moisture Characteristics to the Physical Properties of Soils. Water Resources Research.

[21] Saxton K, Rawls W (2006). Soil Water Characteristic Estimates by Texture and Organic Matter for Hydrologic Solutions. Soil Science Society of America Journal.

[22] Ahuja L, Naney J, Green R, Nielsen D (1984). Macroporosity to characterize spatial variability of hydraulic conductivity and effects of land management. Soil Science Society of America Journal.

[23] Pachepsky Y, Park Y (2015). Saturated Hydraulic Conductivity of US Soils Grouped According to Textural Class and Bulk Density. Soil Science Society of America Journal.

[24] Yao R (2015). Evaluation of pedotransfer functions for estimating saturated hydraulic conductivity in coastal salt-affected mud farmland. Journal of Soils and Sediments.

[25] Vereecken H, Maes J, Feyen J (1990). Estimating unsaturated hydraulic conductivity from easily measured soil properties. Soil Science.

[26] Rawls W, Brakensiek D, Saxton K (1982). Estimation of soil water properties. Trans. Asae.

[27] Lado M, Paz A, Ben-Hur M (2004). Organic Matter and Aggregate-Size Interactions in Saturated Hydraulic Conductivity Contribution from the Agricultural Research Organization, the Volcani Center, no. 623/02, 2002 series. Soil Science Society of America Journal.

[28] Chapuis R (2012). Predicting the saturated hydraulic conductivity of soils: a review. Bulletin of Engineering Geology and the Environment.

[29] Jorda H, Bechtold M, Jarvis N, Koestel J (2015). Using boosted regression trees to explore key factors controlling saturated and near-saturated hydraulic conductivity. European Journal of Soil Science.

[30] Zhang Y, Chen H, Taylor A (2014). Multiple drivers of plant diversity in forest ecosystems. Global Ecology and Biogeography.

[31] De’Ath G (2007). Boosted trees for ecological modeling and prediction. Ecology.

[32] De’Ath G, Fabricius K (2000). Classification and regression trees: a powerful yet simple technique for ecological data analysis. Ecology.

[33] Gonzalez R, Cooperband L (2002). Compost Effects on Soil Physical Properties And Field Nursery Production. Compost Science & Utilization.

[34] Hudson B (1994). Soil organic matter and available water capacity. Journal of Soil and Water Conservation.

[35] Keller T, Sutter J, Nissen K, Rydberg T (2012). Using field measurement of saturated soil hydraulic conductivity to detect low-yielding zones in three Swedish fields. Soil and Tillage Research.

[36] Bodner G, Loiskandl W, Buchan G, Kaul H (2008). Natural and management-induced dynamics of hydraulic conductivity along a cover-cropped field slope. Geoderma.

[37] Chaves J (2008). Land management impacts on runoff sources in small Amazon watersheds. Hydrological Processes.

[38] Jarvis N, Koestel J, Messing I, Moeys J, Lindahl A (2013). Influence of soil, land use and climatic factors on the hydraulic conductivity of soil. Hydrology and Earth System Sciences.

[39] Rasse D, Smucker A, Santos D (2000). Alfalfa Root and Shoot Mulching Effects on Soil Hydraulic Properties and Aggregation. Soil Science Society of America Journal.

[40] Wilcox B, Breshears D, Turin H (2003). Hydraulic Conductivity in a Piñon-Juniper Woodland. Soil Science Society of America Journal.

[41] Brassard B, Chen H, Bergeron Y, Paré D (2011). Differences in fine root productivity between mixed- and single-species stands. Functional Ecology.

[42] Vandevivere P, Baveye P (1992). Effect of bacterial extracellular polymers on the saturated hydraulic conductivity of sand columns. Applied and Environmental Microbiology.

[43] Marschner P, Kandeler E, Marschner B (2003). Structure and function of the soil microbial community in a long-term fertilizer experiment. Soil Biology & Biochemistry.

[44] Song Z, Wang F, Lin H, Mu X, Kang S (2011). Simulation experiment of roots system effects on soil mechanical pressure. Transactions of the Chinese Society of Agricultural Engineering.

[45] Bird J, Torn M (2006). Fine roots vs. needles: a comparison of 13 C and 15 N dynamics in a ponderosa pine forest soil. Biogeochemistry.

[46] Grayston S, Griffith G, Mawdsley J, Campbell C, Bardgett R (2001). Accounting for variability in soil microbial communities of temperate upland grassland ecosystems. Soil Biology and Biochemistry.

[47] Nsabimana D, Haynes R, Wallis F (2004). Size, activity and catabolic diversity of the soil microbial biomass as affected by land use. Applied Soil Ecology.

[48] Shah Z, Adamst W, Haven C (1990). Composition and activity of the microbial population in an acidic upland soil and effects of liming. Soil Biology and Biochemistry.

[49] Branham J, Strack M (2014). Saturated hydraulic conductivity in Sphagnum‐dominated peatlands: do microforms matter?. Hydrological Processes.

[50] Chamley, F., Hardie, M., Lambert, S. & Doyle, R. In *5th Joint Soil Science Australia and New Zealand Society of Soil Science Conference*. 221–224.

[51] Aggelides S, Londra P (2000). Effects of compost produced from town wastes and sewage sludge on the physical properties of a loamy and a clay soil. Bioresource technology.

[52] Benjamin J, Mikha M, Vigil M (2008). Organic carbon effects on soil physical and hydraulic properties in a semiarid climate. Soil Science Society of America Journal.

[53] Fontaine, S. *et al*. Stability of organic carbon in deep soil layers controlled by fresh carbon supply. *Nature* **450**, 277–280, doi:nature06275 (2007). 10.1038/nature06275 17994095

[54] García-Orenes F (2005). Factors controlling the aggregate stability and bulk density in two different degraded soils amended with biosolids. Soil and Tillage Research.

[55] Wang T, Wedin D, Zlotnik V (2009). Field evidence of a negative correlation between saturated hydraulic conductivity and soil carbon in a sandy soil. Water Resources Research.

[56] Carter, M. & Stewart, B. *Structure and organic matter storage in agricultural soils*. Vol. 8 (CRC press, 1995).

[57] Hackl E, Pfeffer M, Donat C, Bachmann G, Zechmeister-Boltenstern S (2005). Composition of the microbial communities in the mineral soil under different types of natural forest. Soil Biology and Biochemistry.

[58] Chen H, Shrestha B (2012). Stand age, fire and clearcutting affect soil organic carbon and aggregation of mineral soils in boreal forests. Soil Biology and Biochemistry.

[59] Cusack D, Chadwick O, Ladefoged T, Vitousek P (2013). Long-term effects of agriculture on soil carbon pools and carbon chemistry along a Hawaiian environmental gradient. Biogeochemistry.

[60] Helfrich M, Ludwig B, Buurman P, Flessa H (2006). Effect of land use on the composition of soil organic matter in density and aggregate fractions as revealed by solid-state 13C NMR spectroscopy. Geoderma.

[61] Laganière J (2013). Stability of Soil Carbon Stocks Varies with Forest Composition in the Canadian Boreal Biome. Ecosystems.

[62] Faithfull, N. T. Methods in agricultural chemical analysis: a practical handbook. (CABI Publishing, 2002).

[63] Nimmo J (2004). Porosity and pore size distribution. Encyclopedia of Soils in the Environment.

[64] Li Y, Shao M (2006). Change of soil physical properties under long-term natural vegetation restoration in the Loess Plateau of China. Journal of Arid Environments.

[65] ISSAS. 515–517 (Shanghai Science and Technology Press, Shanghai, China, 1978).

[66] Wang Y, Zhang J, Zhang Z (2015). Influences of intensive tillage on water-stable aggregate distribution on a steep hillslope. Soil and Tillage Research.

[67] Bates, D., Maechler, M., Bolker, B. & Walker, S. lme4: Linear mixed-effects models using Eigen and S4. *R package version* **1** (2014).

[68] Kuznetsova, A., Brockhoff, P. & Christensen, R. Package ‘lmerTest’. *R package version* **2** (2015).

[69] Box, G. & Cox, D. An analysis of transformations. *Journal of the Royal Statistical Society. Series B (Methodological)*, 211–252 (1964).

[70] Ridgeway G, Southworth M, RUnit S (2013). Package ‘gbm’. Viitattu.

