# Supplementary material for: Impacts of changes in vegetation on saturated hydraulic conductivity of soil in subtropical forests
Source: Sci Rep. 2019 Jun 10;9:8372. doi: 10.1038/s41598-019-44921-w (PMC6557890; doi:10.1038/s41598-019-44921-w)
Supplement: Supplementary file 1 — Supplementary information [file 41598_2019_44921_MOESM1_ESM.docx]

# Impacts of changes in vegetation on saturated hydraulic conductivity of soil in subtropical forests

Mingzhuo Hao^1^, Jinchi Zhang^1, *^, Miaojing Meng^1^, Han Y. H. Chen^2^,

Xiaoping Guo^1, 3, *^, Shenglong Liu^1, 4^, Lixin Ye ^4^

^1^ Jiangsu Province Key Laboratory of Soil and Water Conservation and Ecological Restoration, Co-Innovation Center for the Sustainable Forestry in Southern China, College of Forestry, Nanjing Forestry University, 159 Longpan Road, Nanjing, Jiangsu 210037, China

^2^ Faculty of Natural Resource Management, Lakehead University, 955 Oliver Road, Thunder Bay, Ontario P7B 5E1, Canada

^3^ Nanjing Institute of Environmental Sciences, Ministry of Ecology and Environment, 8 Jiangwangmiao Street, Nanjing Jiangsu 210042, China

^4^ Feng yang Mountain Administration of Zhejiang Feng yang Mountain-Baishanzu National Nature Reserve, 55 Zhongshan West Road, Longquan, Zhejiang 323700, China

*Corresponding authors:

Email: zhangjc8811@sohu.com (Jinchi Zhang), phone: +86-25-8542-7097

Email: gxp@nies.org (Xiaoping Guo), phone & fax: +86-25-8528-7290.

# Date sets

**Part 1 Soil characteristics**

| Plot | LD | LAY | pH | TN | TP | BD | TOC | SAT | CAP | NCAP | A1 | A2 | A3 | K |
| --- | --- | --- | --- | --- | --- | --- | --- | --- | --- | --- | --- | --- | --- | --- |
| 1 | A | a | 5.31 | 4.28 | 0.57 | 1.18 | 31.46 | 55.47 | 47 | 8.47 | 49 | 40 | 11 | 2.2 |
| 1 | A | b | 5.44 | 4.17 | 0.52 | 1.39 | 22.75 | 47.55 | 43 | 4.55 | 55 | 27 | 18 | 2.51 |
| 1 | A | c | 4.94 | 4.48 | 0.5 | 1.39 | 22.8 | 47.55 | 46 | 1.55 | 40 | 39 | 21 | 3.01 |
| 2 | A | a | 5.49 | 5.3 | 0.44 | 1.24 | 49.65 | 53.21 | 50 | 3.21 | 62 | 34 | 4 | 1.83 |
| 2 | A | b | 4.98 | 4.81 | 0.53 | 1.31 | 42.87 | 50.57 | 41 | 9.57 | 34 | 39 | 26 | 2.57 |
| 2 | A | c | 5.25 | 4.68 | 0.56 | 1.53 | 41.7 | 42.26 | 29 | 13.26 | 42 | 36 | 22 | 1.37 |
| 3 | A | a | 5.22 | 4.23 | 0.58 | 1.09 | 45.24 | 58.87 | 51 | 7.87 | 50 | 49 | 1 | 1.83 |
| 3 | A | b | 4.96 | 3.96 | 0.72 | 1.21 | 38.71 | 54.34 | 42 | 12.34 | 51 | 35 | 14 | 2.81 |
| 3 | A | c | 5.33 | 6.84 | 0.68 | 1.51 | 21.08 | 43.02 | 35 | 8.02 | 41 | 34 | 25 | 2.38 |
| 4 | A | a | 5.92 | 4.87 | 0.68 | 1.22 | 26.31 | 53.96 | 49 | 4.96 | 38 | 52 | 10 | 2.57 |
| 4 | A | b | 6.18 | 5.09 | 0.54 | 1.38 | 20.05 | 47.92 | 42 | 5.92 | 69 | 20 | 11 | 2.32 |
| 4 | A | c | 6.95 | 5.16 | 0.67 | 1.39 | 13.04 | 47.55 | 42 | 5.55 | 61 | 21 | 18 | 3.06 |
| 5 | A | a | 5.12 | 5.23 | 0.49 | 1.28 | 39.27 | 51.7 | 43 | 8.7 | 43 | 38 | 20 | 1.35 |
| 5 | A | b | 5.05 | 4.92 | 0.51 | 1.41 | 29.14 | 46.79 | 41 | 5.79 | 78 | 11 | 11 | 2.57 |
| 5 | A | c | 5.67 | 5.18 | 0.56 | 1.57 | 27.23 | 40.75 | 29 | 11.75 | 38 | 42 | 20 | 2.87 |
| 6 | B | a | 5.28 | 2.74 | 0.38 | 1.52 | 23.34 | 42.64 | 33 | 9.64 | 34 | 46 | 20 | 1.71 |
| 6 | B | b | 4.91 | 1.25 | 0.65 | 1.61 | 20.17 | 39.25 | 34 | 5.25 | 42 | 41 | 17 | 0.58 |
| 6 | B | c | 5.74 | 2.1 | 0.68 | 1.61 | 14.81 | 39.25 | 28 | 11.25 | 32 | 39 | 29 | 1.65 |
| 7 | B | a | 4.99 | 1.99 | 0.42 | 1.23 | 20.24 | 53.58 | 47 | 6.58 | 36 | 47 | 17 | 2.26 |
| 7 | B | b | 4.52 | 1.92 | 0.58 | 1.4 | 16.45 | 47.17 | 39 | 8.17 | 46 | 46 | 9 | 0.73 |
| 7 | B | c | 5.52 | 1.37 | 0.53 | 1.6 | 13.8 | 39.62 | 29 | 10.62 | 41 | 40 | 19 | 1.19 |
| 8 | B | a | 5.18 | 2.79 | 0.51 | 1.31 | 34.57 | 50.57 | 45 | 5.57 | 65 | 30 | 6 | 2.26 |
| 8 | B | b | 5.17 | 3.46 | 0.5 | 1.39 | 33.24 | 47.55 | 41 | 6.55 | 52 | 34 | 13 | 0.81 |
| 8 | B | c | 4.95 | 3.8 | 0.43 | 1.44 | 29.03 | 45.66 | 37 | 8.66 | 43 | 40 | 17 | 1.71 |
| 9 | B | a | 4.96 | 4.58 | 0.44 | 1.53 | 60.11 | 42.26 | 33 | 9.26 | 42 | 38 | 20 | 1.96 |
| 9 | B | b | 5.12 | 4.12 | 0.51 | 1.59 | 29.38 | 40 | 28 | 12 | 42 | 42 | 16 | 0.85 |
| 9 | B | c | 5.58 | 4.29 | 0.52 | 1.74 | 28.92 | 34.34 | 29 | 5.34 | 50 | 32 | 17 | 0.85 |
| 10 | B | a | 6.12 | 3.75 | 0.51 | 1.58 | 45.21 | 40.38 | 38 | 2.38 | 63 | 23 | 13 | 1.22 |
| 10 | B | b | 5.03 | 3.79 | 0.47 | 1.58 | 28.37 | 40.38 | 29 | 11.38 | 52 | 34 | 14 | 1.13 |
| 10 | B | c | 4.96 | 3.8 | 0.61 | 1.73 | 11.89 | 34.72 | 14 | 20.72 | 61 | 32 | 6 | 1.9 |
| 11 | C | a | 5.77 | 4.12 | 0.59 | 1.09 | 38.24 | 58.87 | 50 | 8.87 | 50 | 40 | 10 | 2.81 |
| 11 | C | b | 5.04 | 5 | 0.52 | 1.26 | 19.25 | 52.45 | 49 | 3.45 | 44 | 39 | 17 | 1.71 |
| 11 | C | c | 5.57 | 3.36 | 0.48 | 1.52 | 25.52 | 42.64 | 37 | 5.64 | 52 | 26 | 22 | 2.08 |
| 12 | C | a | 6.01 | 3.38 | 0.54 | 1.05 | 40.52 | 60.38 | 51 | 9.38 | 33 | 42 | 24 | 2.57 |
| 12 | C | b | 5.28 | 2.63 | 0.56 | 1.14 | 29.34 | 56.98 | 45 | 11.98 | 40 | 47 | 14 | 1.25 |
| 12 | C | c | 4.88 | 2.72 | 0.61 | 1.26 | 25.66 | 52.45 | 47 | 5.45 | 41 | 40 | 19 | 1.71 |
| 13 | C | a | 5.69 | 4.12 | 0.47 | 1.21 | 39.61 | 54.34 | 46 | 8.34 | 61 | 36 | 4 | 2.81 |
| 13 | C | b | 5.71 | 3.33 | 0.57 | 1.32 | 30.19 | 50.19 | 44 | 6.19 | 52 | 29 | 19 | 0.95 |
| 13 | C | c | 5.58 | 4.49 | 0.52 | 1.34 | 28.18 | 49.43 | 40 | 9.43 | 52 | 33 | 14 | 2.45 |
| 14 | C | a | 5.69 | 2.25 | 0.73 | 1.31 | 28.34 | 50.57 | 41 | 9.57 | 44 | 47 | 9 | 1.71 |
| 14 | C | b | 6.31 | 1.96 | 0.43 | 1.4 | 20.27 | 47.17 | 39 | 8.17 | 42 | 45 | 13 | 1.59 |
| 14 | C | c | 6.36 | 2.45 | 0.4 | 1.4 | 18.02 | 47.17 | 38 | 9.17 | 39 | 46 | 15 | 1.83 |
| 15 | C | a | 6.35 | 1.34 | 0.41 | 1.26 | 29.98 | 52.45 | 43 | 9.45 | 40 | 45 | 15 | 2.94 |
| 15 | C | b | 6.21 | 1.21 | 0.49 | 1.28 | 20.17 | 51.7 | 41 | 10.7 | 45 | 38 | 17 | 0.8 |
| 15 | C | c | 5.95 | 1.11 | 0.54 | 1.39 | 11.41 | 47.55 | 34 | 13.55 | 44 | 41 | 16 | 3.67 |
| 16 | D | a | 5.02 | 1.38 | 0.48 | 1.21 | 29.33 | 54.34 | 46 | 8.34 | 54 | 29 | 17 | 2.2 |
| 16 | D | b | 5.19 | 1.59 | 0.51 | 1.28 | 23.15 | 51.7 | 45 | 6.7 | 51 | 42 | 7 | 1.13 |
| 16 | D | c | 5.15 | 1.71 | 0.6 | 1.35 | 25.19 | 49.06 | 44 | 5.06 | 56 | 32 | 13 | 1.44 |
| 17 | D | a | 5.31 | 2.87 | 0.44 | 1.18 | 50.23 | 55.47 | 41 | 14.47 | 47 | 40 | 13 | 3.42 |
| 17 | D | b | 4.98 | 2.92 | 0.51 | 1.19 | 25.17 | 55.09 | 49 | 6.09 | 45 | 33 | 22 | 0.54 |
| 17 | D | c | 4.83 | 2.7 | 0.79 | 1.29 | 19.49 | 51.32 | 45 | 6.32 | 49 | 35 | 16 | 1.31 |
| 18 | D | a | 5.12 | 2.19 | 0.7 | 1.19 | 38.71 | 55.09 | 43 | 12.09 | 59 | 31 | 10 | 2.94 |
| 18 | D | b | 5.09 | 3.37 | 0.5 | 1.28 | 35.38 | 41.7 | 34 | 7.7 | 62 | 30 | 8 | 0.4 |
| 18 | D | c | 4.46 | 3.77 | 0.63 | 1.52 | 16.78 | 42.64 | 32 | 10.64 | 43 | 37 | 20 | 1.32 |
| 19 | D | a | 6.28 | 2.01 | 0.53 | 1.11 | 33.74 | 58.11 | 52 | 6.11 | 45 | 36 | 19 | 1.47 |
| 19 | D | b | 5.17 | 1.99 | 0.35 | 1.22 | 28.17 | 53.96 | 51 | 2.96 | 48 | 37 | 15 | 0.82 |
| 19 | D | c | 5.56 | 2.27 | 0.59 | 1.42 | 12.97 | 46.42 | 37 | 9.42 | 30 | 44 | 25 | 2.43 |

LD – vegetation type where A – native forest, B – mixed forest, C – bamboo forest, D – tea garden. LAY – soil depth, where a – 0-10cm, b – 10-20cm, c – 20-30cm. TN – total nitrogen concentration (g kg^-1^). TP – total phosphorus concentration (g kg^-1^). BD – bulk density. (Mg m^-3^). TOC – total organic carbon ((g kg^-1^). SAT –total porosity (%). CAP – capillary porosity (%). NCAP – non-capillary porosity (%). A1 – Macro water-stable aggregate (%). A2 – Meso water-stable aggregate (%). A3 – Micro water-stable aggregate (%). K – *K_s_* (m day^-1^).

**Part 2 Root characteristics**

| Plot | LD | LAY | RLD | RSAD |
| --- | --- | --- | --- | --- |
| 1 | A | a | 4.89 | 49.6 |
| 1 | A | b | 4.46 | 40.6 |
| 1 | A | c | 3.95 | 39.4 |
| 2 | A | a | 3.72 | 60.7 |
| 2 | A | b | 3.35 | 61.8 |
| 2 | A | c | 3.94 | 22.8 |
| 3 | A | a | 4.4 | 68.2 |
| 3 | A | b | 3.43 | 22.1 |
| 3 | A | c | 1.98 | 28.2 |
| 4 | A | a | 4.09 | 55.4 |
| 4 | A | b | 3.36 | 40.2 |
| 4 | A | c | 3.14 | 22.3 |
| 6 | A | a | 4.18 | 59.3 |
| 6 | A | b | 3.16 | 49.7 |
| 6 | A | c | 4.6 | 28.2 |
| 7 | B | a | 4.02 | 62.19 |
| 7 | B | b | 1.26 | 13.42 |
| 7 | B | c | 3.57 | 21.7 |
| 8 | B | a | 5.45 | 87.5 |
| 8 | B | b | 4.14 | 25.9 |
| 8 | B | c | 3.81 | 24.5 |
| 9 | B | a | 5.32 | 50.5 |
| 9 | B | b | 3.56 | 22.9 |
| 9 | B | c | 4.32 | 25.2 |
| 10 | B | a | 3.96 | 46.1 |
| 10 | B | b | 3.98 | 68.7 |
| 10 | B | c | 3.86 | 44.1 |
| 11 | C | a | 8.92 | 35.34 |
| 11 | C | b | 7.94 | 34.84 |
| 11 | C | c | 6.6 | 29.76 |
| 12 | C | a | 8.59 | 35.34 |
| 12 | C | b | 6.38 | 30.16 |
| 12 | C | c | 4.35 | 23.77 |
| 13 | C | a | 8.12 | 34.43 |
| 13 | C | b | 6.08 | 29.76 |
| 13 | C | c | 5.71 | 27.93 |
| 14 | D | a | 3.76 | 22.65 |
| 14 | D | b | 2.76 | 18.59 |
| 14 | D | c | 3.55 | 20.52 |
| 15 | D | a | 4.41 | 25.49 |
| 15 | D | b | 2.02 | 18.38 |
| 15 | D | c | 1.02 | 14.23 |
| 16 | D | a | 4.03 | 24.17 |
| 16 | D | b | 3.1 | 19.3 |
| 16 | D | c | 1.39 | 14.12 |

LD – vegetation type where A – native forest, B – mixed forest, C – bamboo forest, D – tea garden. LAY – soil depth, where a – 0-10cm, b – 10-20cm, c – 20-30cm. RLD – Root length density (cm cm^-3^). RSAD – Root surface area density (cm^2^ cm^-3^).

# Supplementary Figures

**Forests and root photos**

**
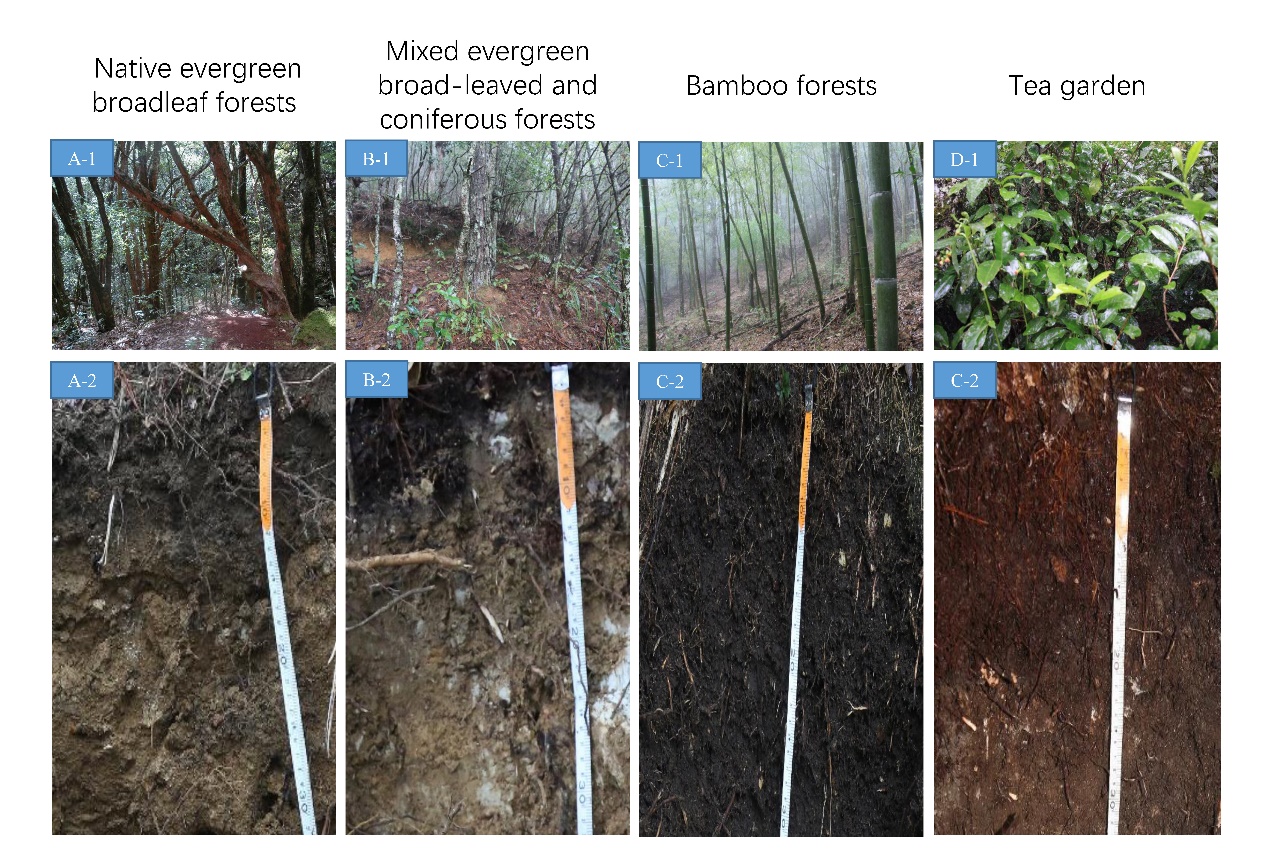
**

Photos were taken by Shenglong Liu.
